# Army and Navy Notes

**Published:** 1898-07

**Authors:** 


### ARMY AND NAVY NOTES.

The following assignments of chief medical officers of the army corps have been announced :

To 1st army corps, Lieut.-Col. Rush Huidekoper, U. S. V.
To 2d army corps, Lieut-Col. Alfred C. Girard, U. S. V.
To 3d army corps, Lieut-Col. John Van R. Hoff, U. S. V.
To 4th army corps, Lieut.-Col. Robert M. O'Reilly, U. S. V.
To 5th army corps, Lieut-Col. Benjamin F. Pope, U. S. V.
To 6th army corps, Lieut.-Col. Nicholas Senn, U. S. V.
To 7th army corps, Lieut.-Col. Louis M. Maus, U. S. V.

Appointments to the medical staff of the army have been announced as follows :

To be chief surgeons of division with rank of major : Captain William H. Arthur, assistant suigeon U. S. A., Captain George E. Bushnell, assistant surgeon U. S. A.; Donald Maclean, of Michigan ; George Ryerson Fowler, of New York.

Captains and assistant surgeons U. S. Army to be brigade surgeons, with the rank of major :

William C. Gorgas,

Henry P. Birmingham,

M. C. Wyeth,

Richard W. Johnson,

Edward C. Carter,

William O. Owen,

Peter G. Regan,

William J. Wakeman,

William Stephenson,

Adrian S. Polhemus,

John L. Phillips,

William C. Borden,

Edgar A. Meams,

Guy L. Edie,

William D. Crosby,

William L. Kneedler,

Charles M. Gandy,

James E. Pilcher,

Charles B. Ewing,

Walter D. M'Caw,

Jefferson R. Kean,

Henry I. Raymond,

Francis J. Ives,

William P. Kendall,

Edward R. Morris,

Henry S. Harris,

William B. Bannister,

Paul Clendennin,

Charles E. Woodruff,

Eugene L. Swift,

Paul S. Hillock,

Ogden Rafferty,

Charles F. Mason,

James D. Glennan.

Alfred E. Bradly,

Philip G. Wales.

To be brigade surgeons from civil life with the rank of major : Willis G. Macdonald, of New York ; Charles M. Drake, of Georgia ; Joseph K. Weaver, of Pennsylvania ; John Guiteras, of Pennsylvania ;

Charles E. Ruth, of Iowa; John W. Bayne, of the District of Columbia; Milo B. Ward, of Missouri; Schuyler C. Graves, of Michigan; George T. Vaughan, of Marine Hospital Service; Nathan S. Jarvis, of New York; William DeVine, of Massachusetts; John C. Martin, of Ohio; Peter D. MacNaughton, of Michigan; Samuel T. Armstrong, acting assistant surgeon; John Patterson Dodge, of Ohio; John R. M'Dill, of Wisconsin; Samuel O. L. Potter, of California; George A. Smith, of Iowa; Arthur Snowden, of Virginia; R. Stansbury Sutton, of Pennsylvania; C. Frank Bruso, of New York; George W. Crile, of Ohio; Edward Martin, of Pennsylvania; Calvin H. English, of Indiana; George B. Bunn, of Ohio; George H. Penrose, of Utah; Ernest Taylor Tappey, of Michigan.

---

A COMPARISON of the cost of the army and navy hospital ships is interesting at this time as showing the care the government takes of its soldiers and sailors when sick.

More than $1,000,000 has been paid for the two hospital ships, the Solace and the Relief, $450,000 of which to the Maine Steamship Company for the John Englis, which has been renamed the Relief, and is being fitted up by the war department as a floating hospital.

The price paid to the Cromwell Line for the Creole, which is now the Solace, was $600,000, making a total of $1,050,000 for the two hospital vessels. The estimated cost to the war department for fitting up the Relief is $50,000, in addition to many things that are to be presented by various organisations, which are working to aid in reducing the horrors of war as much as possible. It has been stated that about twice as much has been expended in fitting up the Solace, as the navy department had a special appropriation for the purpose and also greater facilities for doing work upon ships, as it controlled the navy yards. The total expense of procuring and equipping the two vessels will not be far from $1,250,000.

A TRAIN of hospital cars has been organised by Surgeon-General Sternberg which is now located in Florida, where it will be used as a hospital for the camp. When there are as many patients on board as it can comfortably hold, or when the patients' removal from that climate is thought to be advisable, the train will be sent to Atlanta, there to deposit the sick in the general hospital which has been established at Fort McPherson. There are branch hospitals at Fort Thomas, Ky., and at Chickamauga, and between all these points the train will be run whenever necessary to insure the welfare of sick or wounded men. The train consists of two dining cars and ten sleeping coaches. The cars are of the tourist kind which are used in Europe. They are as comfortable, but not so elegant as the regular Pullman sleeper. The train is equipped with all necessaries and appliances, and a staff of army surgeons is attached to it.

---

THE governor of Pennsylvania, himself a soldier, showed his appreciation of the importance of sending forward properly equipped medical officers with the troops, by appointing two of the best-known physicians of the Keystone state to serve with an army surgeon as a board of examination, to pass upon the professional fitness and standing of all medical officers commissioned by Governor Hastings. The civilian appointees were Dr. William Pepper, of Philadelphia, and Dr. William S. Foster, of Pittsburg. The board has completed its work, having recommended fifty-five candidates for positions as surgeons and assistant surgeons of Pennsylvania volunteers. What a pity New York did not adopt a similar plan!

---

THE hospital ship Relief, formerly the John Englis, is pieparing to sail. A few days ago the staff was ordered to muster at the vessel and form an officers mess.

The members are Dr. George H. Torney, major and surgeon-in-chief; Dr. W. C. Gorgas, major and assistant surgeon; Drs. Francis T. Metcalfe, of Buffalo, Randolph M. Meyers, of Washington, and Llewellyn P. Williamson, of St. Louis, captains and assistant surgeons; Dr. William M. Gray, of the Army Medical Museum, at Washington, microscopist, and Drs. Ernest C. Schultz, of New York, Thomas A. Smith, of New York, and Frederick McG. Hartsook, of Baltimore, acting assistant surgeons.

---

MRS. L. Z. LEITER, of Chicago, has presented to the government a hospital for military use at Camp Thomas. It is the building known as Chickamauga Park Hotel, at Crawfish Springs, and cost about $60,000 besides repairs. This patriotic act deserves the commendation of all men. The hospital has accommodations for 300 or 400 patients and will be, when finished, the most complete general hospital yet established with troops in the field.

---

ST. LUKE'S hospital, New York, is to have a soldiers' ward. The trustees lately met at the hospital and approved of the action of the executive committee in setting aside a ward in the institution for the exclusive use during the war of soldiers and sailors who may need medical or surgical aid. Letters were read from the surgeon-general of the navy and from the surgeon-general of the army accepting the offer and thanking the hospital for making it.

